# Novel Antibodies Reveal Inclusions Containing Non-Native SOD1 in Sporadic ALS Patients

**DOI:** 10.1371/journal.pone.0011552

**Published:** 2010-07-14

**Authors:** Karin Forsberg, P. Andreas Jonsson, Peter M. Andersen, Daniel Bergemalm, Karin S. Graffmo, Magnus Hultdin, Johan Jacobsson, Roland Rosquist, Stefan L. Marklund, Thomas Brännström

**Affiliations:** 1 Department of Medical Biosciences, Umeå University, Umeå, Sweden; 2 Department of Pathology, Umeå University, Umeå, Sweden; 3 Department of Clinical Chemistry, Umeå University, Umeå, Sweden; 4 Department of Pharmacology and Clinical Neuroscience, Umeå University, Umeå, Sweden; 5 Department of Molecular Biology, Umeå University, Umeå, Sweden; Uppsala University, Sweden

## Abstract

Mutations in CuZn-superoxide dismutase (SOD1) cause amyotrophic lateral sclerosis (ALS) and are found in 6% of ALS patients. Non-native and aggregation-prone forms of mutant SOD1s are thought to trigger the disease. Two sets of novel antibodies, raised in rabbits and chicken, against peptides spaced along the human SOD1 sequence, were by enzyme-linked immunosorbent assay and an immunocapture method shown to be specific for denatured SOD1. These were used to examine SOD1 in spinal cords of ALS patients lacking mutations in the enzyme. Small granular SOD1-immunoreactive inclusions were found in spinal motoneurons of all 37 sporadic and familial ALS patients studied, but only sparsely in 3 of 28 neurodegenerative and 2 of 19 non-neurological control patients. The granular inclusions were by confocal microscopy found to partly colocalize with markers for lysosomes but not with inclusions containing TAR DNA binding protein-43, ubiquitin or markers for endoplasmic reticulum, autophagosomes or mitochondria. Granular inclusions were also found in carriers of SOD1 mutations and in spinobulbar muscular atrophy (SBMA) patients and they were the major type of inclusion detected in ALS patients homozygous for the wild type-like D90A mutation. The findings suggest that SOD1 may be involved in ALS pathogenesis in patients lacking mutations in the enzyme.

## Introduction

Amyotrophic lateral sclerosis (ALS) is a fatal neurodegenerative syndrome characterized by adult-onset progressive loss of motoneurons in the cortex, brain stem and ventral horns of the spinal cord. Approximately 10% of ALS patients are familial (FALS) [1] and in 12–23% of these the disease has been linked to mutations in the gene for CuZn-superoxide dismutase (SOD1) [2]. SOD1 is ubiquitously expressed and the mutations confer an unidentified toxic property on the enzyme [3], [4], [5]. SOD1 mutations have also been found in apparently sporadic ALS (SALS) patients and overall, they are detected in about 6% of all ALS patients [6]. The cause(s) of the disease in the remainder is largely unknown. In several other neurodegenerative conditions such as Alzheimer's, Parkinson's and Creutzfeldt-Jacob's diseases, proteins that are mutated in some of the familial patients are also thought to be involved in the pathogenesis in patients lacking such mutations [7]. Could wild-type SOD1, by analogy, be involved in ALS patients lacking SOD1 mutations?

The toxic property of mutant SOD1s has not been identified, but there is evidence to suggest that it is related to structural instability and noxious effects of non-native, misfolded and aggregation-prone conformational species of SOD1 [8], [9], [10], [11], [12]. The 146 ALS-associated mutant SOD1s identified to date [6] cover a spectrum from extreme instability to near wild type-like stability *in vivo* in humans [4], [9], [13], [14]. The most wild type-like mutant SOD1 (D90A) is found at normal levels in the CNS of ALS patients homozygous for the mutation [15]. There are indications that wild-type human SOD1 can also be toxic. Overexpression in transgenic mice leads to a substantial late loss of neurons in the spinal cord ventral horns [16], [17] and exacerbates disease caused by mutant SOD1s [17], [18]. Post-translational modifications of wild-type SOD1, e.g. by oxidative insults, can destabilize the enzyme [19] and induce neurotoxic properties [20]. Crosslinked SOD1 can be detected in extracts of spinal cord tissue from both carriers of SOD1 mutations and SALS cases, but not from controls [21]. Thus, there is circumstantial evidence to suggest that the wild-type SOD1 has the potential to exert ALS-causing noxious effects similar to those of mutant SOD1s.

To explore this idea further, we produced two sets of antibodies (in rabbits [Ra-ab] and chicken [Ch-ab]) directed against peptides spaced along the sequence of the SOD1 molecule. These were used to look for evidence of SOD1 alterations in ALS patients without SOD1 mutations. By biochemical methods we showed that these antibodies were specific for denatured SOD1. Using both histopathological and biochemical methods, we examined different areas of the CNS from a large number of sporadic and familial ALS patients and in two motoneuron disease patients with spinobulbar muscular atrophy (SBMA). The main novel finding is that these antibodies detected inclusions, which are considered to be hallmarks of disease caused by mutant SOD1s, in all these patients but rarely in controls with other and without neurodegenerative diseases.

## Results

### Inclusions containing misfolded SOD1 in motoneurons are a feature of both sporadic and familial motoneuron disease

**Figure 1:**
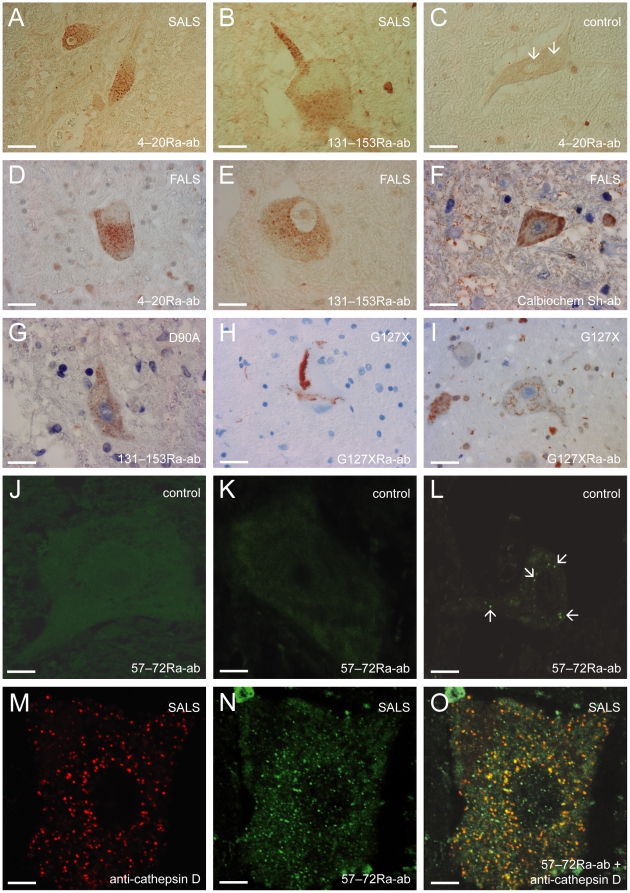
Micrographs of spinal cord motoneurons showing SOD1-immunoreactive inclusions. Using the 4–20Ra-ab, 57–72Ra-ab, and 131–153Ra-ab anti-SOD1 peptide antibodies (0.64, 5 and 0.75 µg/ml, respectively) numerous small granular inclusions could be seen in tissues from sporadic (SALS) and familial (FALS) patients lacking mutations in the SOD1 gene (A, B, D, E, J-L, N and O). Note that the lipofuscin do not stain in B. As a rule motoneurons from the controls lacked inclusions (J, K), but in a few cases were a small number of granular inclusions observed (arrows in C, L). Using a sheep anti-SOD1 antibody against whole SOD1 (Calbiochem), SOD1-immunoreactive inclusions could sometimes be discerned against background staining in ALS patients with abundant small granular inclusions (F). In patients carrying the D90A mutation, small granular inclusions were the major type of inclusion (131–153Ra-ab antibody, 0.75 µg/ml) (G). Using a mutation-specific antibody (10 µg/ml) both larger skein and LBHI-like inclusions (H), as well as small granular inclusions (I), could be seen in an ALS patient carrying the G127X mutation. Sections double-labeled with the the lysosomal marker cathepsin D (M and O; red fluorescence) and the 57–72Ra-ab anti-SOD1 antibody (N and O; green fluorescence; 5 µg/ml). The merged picture of the green and red channel scans shows a partial overlap of green and red fluorescence indicating colocalization of SOD1 and lysosomes (O). Scale bar  = 30 µm (in A–D, H, I), 18 µm (in E–G), 7 µm (in J), 8 µm (in K, M-O) and 11 µm (in L).

Using the set of rabbit antibodies raised against peptides in the SOD1 sequence, we found small round inclusions in spinal cord motoneurons of all the 29 sporadic and 8 familial ALS patients lacking mutations in the SOD1 gene and in the 2 SBMA patients (Figures 1A, B, D, E and N) and (Supporting Information Figures S1B, E, H and K, and S2A]. These inclusions were somal and in many cells they were particularly abundant in the axon hillock (Figures 1B and S2A). They were relatively homogenous in size and measured 0.5–3 µm. The proportion of motoneurons exhibiting such inclusions varied from only a few to most of the motoneurons, and they were seen in all spinal cord cross-sections investigated (Table 1). Small granular SOD1-immunoreactive inclusions were also seen in neurons of Clarke's nucleus in 22 of 23 SALS and in 3 of the 5 FALS patients in which the nucleus of Clarke was available for study, and in both SBMA patients. In hematoxylin/eosin-stained sections, some Lewy body-like hyaline inclusions were seen in motoneurons, and in a few patients a small proportion of such structures were immunoreactive for SOD1. No correlation between the disease duration and amount of inclusions could be seen in those patients from whom reliable data on duration were available. Other pathomorphological observations in the patients are reported as in Supporting Information (Results).

**Table 1:** Immunohistochemical findings.

Patients	SALS	FALS	SBMA	Controls		Controls
				Neurodegen.		Non-neurol.
Number of patients with inclusions						
Cervical spinal cord	27 (27)	7 (7)	2 (2)		2 (26)	0 (18)
Thoracic spinal cord	24 (24)	5 (5)	2 (2)		0 (1)	0 (17)
Lumbar spinal cord	17 (17)	4 (4)	2 (2)		1 (3)	2 (19)
Proportion of motoneurons with inclusions						
Cervical spinal cord	2 (1–3)	3 (1–3)	1,2^1^		0 (0–1)	0 (0)
Thoracic spinal cord	2 (1–3)	2 (1–3)	2,2^1^		0 (0)	0 (0)
Lumbar spinal cord	2 (1–3)	2 (1–2)	2,2^1^		0 (0–1)	0 (0–1)

Findings of the immunohistochemical investigation with regard to the 4–20Ra-ab and 131–153Ra-ab anti-SOD1 peptide antibodies. Data for number of patients with inclusions refer to number of patients with inclusions (total number of patients). Data for proportion of motoneurons with inclusions are shown as median (range) referring to a four-tiered semi-quantitative scale (0  =  no neurons with inclusions; 1 =  <25% of the neurons showing inclusions; 2 = 25%–75% of the neurons showing inclusions; 3 =  >75% of the neurons showing inclusions).

1 Individual values of the two SBMA patients.

In most of the control patients, the motoneurons lacked granular SOD1 staining (Figure 1J and K). In 3 out of the 28 control patients with other neurodegenerative diseases (1 with Huntington's disease [HD]; 1 with Parkinson's disease [PD]; 1 with Alzheimer's disease [AD]), and in 2 of the 19 control patients without neurological disease, a few granular SOD1-immunoreactive inclusions were seen in some motoneurons (Figure 1C and L). The abundance of inclusions was, however, smaller than in any of the ALS patients (Table 1). Also, two of these control patients showed a few small granular SOD1-immunoreactive inclusions in the neurons of Clarke's nucleus. In control patients, no granular SOD1 inclusions were seen in cortex or hippocampus (AD patients), striatum (HD patient) or mesencephalon (PD patients).

### SOD1 inclusions are partially colocalized with lysosomes

To gain insight into the subcellular localization of the granular inclusions, we used double-labeling immunohistochemistry with the 57–72Ra-ab anti-SOD1 antibody and antibodies directed against marker proteins for lysosomes, mitochondria and endoplasmatic reticulum. Using confocal laser microscopy, we could show a partial colocalization of SOD1 inclusions with the lysosomal marker cathepsin D in all patients studied (Figure 1O). Colocalization was found in about 25% of all lysosomes, while the proportion of all granular inclusions colocalizing with the lysosomal marker was less than 25%. We could not detect any colocalization of the SOD1 inclusions and the endoplasmic reticulum markers KDEL and GRP-78, the mitochondrial markers mitochondrial Hsp70 and mitochondrial marker, clone SPM198 or in inclusions containing TAR DNA binding protein-43 (TDP-43), the autophagosomes marker MAP1LC3A or ubiquitin (Figures. S1 and S2).

### Antibody specificity; why have the SOD1 inclusions not been observed before in ALS

**Figure 2:**
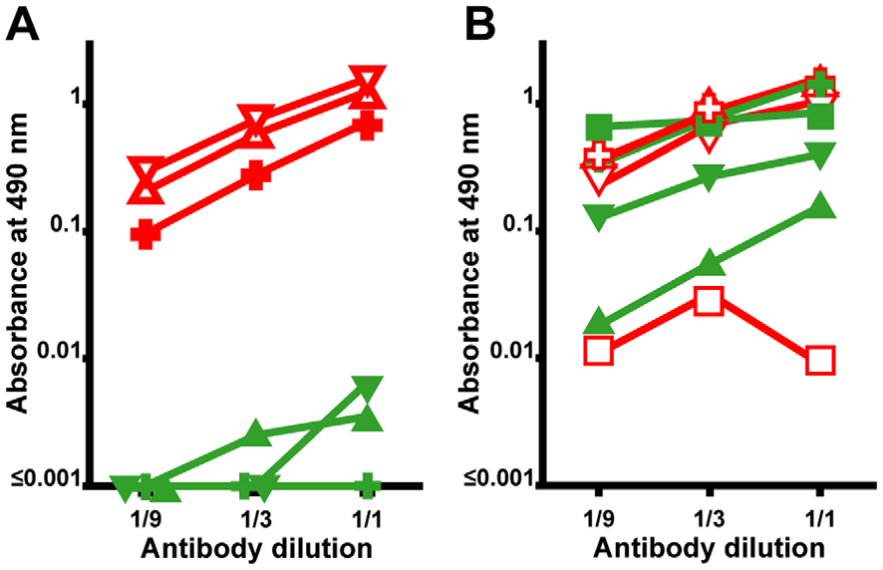
Relative reactivities of antibodies towards native and denatured SOD1. ELISA plates were coated with either native (filled symbols in green) or denatured (unfilled symbols in red) SOD1, and were either reacted with antibodies raised against peptides (A) or whole SOD1 (B). Threefold dilutions were made from a high antibody concentration giving an A_490_ of 0.70–1.6 with either native or denatured SOD1. (A) Reactivity of the 4–20Ra-ab (native  =  ▴, denatured  =  **▵**), 57–72Ra-ab (native SOD1  =  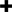, denatured SOD1  =  **“**) and 131–153Ra-ab (native SOD1  =  ▾, denatured SOD1 =  **▿**) anti-SOD1 peptide antibodies. The highest concentrations were 0.1 µg/ml, 0.03 µg/ml, 0.1 µg/ml, respectively. (B) Reactivity of antibodies raised to whole SOD1: Rabbit-1 antibody (native SOD1  =  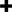;, denatured SOD1  =  **“**); a sheep antibody from Calbiochem (native  =  ▴, denatured  =  **▵**); a sheep antibody from The Binding Site (native SOD1  =  ▾, denatured SOD1 =  **▿**); and a mouse monoclonal antibody from Sigma (native SOD1  =  ▪, denatured SOD1  =  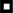). The highest concentrations were 0.6, 10, 20, and 10 µg/ml, respectively. The data presented are means of 4 wells for each point.

In most previous studies, antibodies raised against whole SOD1 have been used [22], [23], [24], [25], [26], [27], [28]. We compared a set of 4 such antibodies with our rabbit anti-SOD1 peptide antibodies for reactivity against denatured and native SOD1 in an ELISA (Figures 2 and S3). As seen in Figure 2A, the 4–20Ra-ab, 57–72Ra-ab and 131–153Ra-ab antibodies reacted strongly with denatured SOD1, but hardly at all with native SOD1. Similar results were found for the other rabbit anti-SOD1 peptide antibodies (Figure S3). Among the antibodies raised against whole SOD1, our in-house rabbit antibody (Rabbit-1) reacted equally to denatured and native SOD1 in the ELISA. The sheep antibodies from the Binding Site and Calbiochem were a little and clearly more, respectively, active versus denatured than against native SOD1 (Figure 2B). The monoclonal mouse antibody was almost completely specific for native SOD1. Most of the antibodies were also immobilized on Sepharose and tested for their affinities for native and denatured SOD1 in solution (Figures S4 and S5). The outcomes were in principle identical; the antipeptide antibodies bound denatured SOD1 but lacked affinity for native, whereas antibodies raised against whole SOD1 bound both native and denatured SOD1.

For comparison purposes we tested dilution series of the set of 4 antibodies raised against whole human SOD1 on sections from 4 patients with abundant small granular inclusions. Using the antibody with the highest relative reactivity for denatured SOD1 (the Calbiochem antibody) (Figure 2B), granular inclusions could sometimes be discerned against the background staining of cytosolic SOD1 (Figure 1F). Thus, the failure in previous studies to detect the granular SOD1 inclusions might be explained the masking effect of staining of the abundant native SOD1 in the motoneurons.

**Figure 3:**
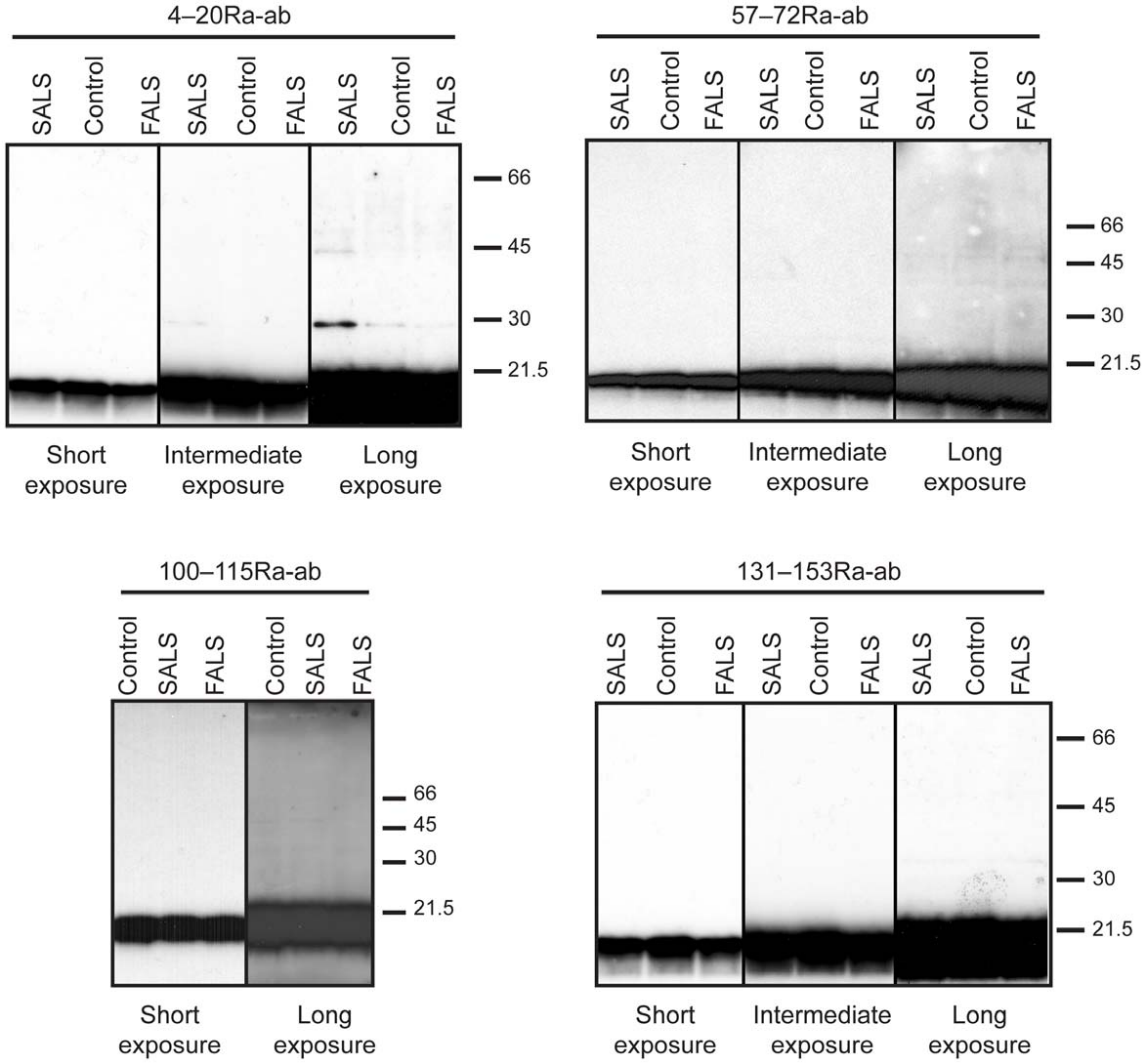
Analysis of SOD1 by western immunoblot. Homogenates of tissue from the temporal lobe, the precentral gyrus, and the spinal cord ventral horns from 5 control patients, 5 SALS and 4 FALS patients were analyzed by western immunoblots, using the 4–20Ra-ab, 57–72Ra-ab, 100–115Ra-ab and 131–153Ra-ab anti-SOD1 peptide antibodies. Analyses of lumbar spinal cord ventral horns are here shown as examples. All figures depict the same set of extracts and short, intermediate and long exposures are presented. Upon long exposure a weak band at about 28 kDa was seen with the 4–20Ra-ab anti-SOD1 antibody in one of the five SALS samples. The band was probably unspecific since it was not seen with the other antibodies. The intensity was estimated at <0.5% of the SOD1 band.

To demonstrate the specificity for SOD1 in the histopathological studies, the antibodies were preincubated with denatured SOD1 (not shown) or the immunizing peptides in increasing concentrations. This resulted in gradual and finally full disappearance of immunoreactivity (Figure S2D, E and F). An antibody raised against keyhole limpet hemocyanin, the carrier protein to which the peptides were coupled for immunization, gave no specific staining. The antibodies stained inclusions of human SOD1 in transgenic mice (Figure S2G), but did not stain tissues from normal control or SOD1 knockout mice (Figure S2H and I). The rabbit peptide antibodies showed very high specificities for SOD1 as analyzed by western immunoblots of human spinal cord extracts (Figure 3). In western blots of extracts of murine spinal cords, there was strong staining for human SOD1 in wild-type human SOD1 transgenic mice, variable weak staining of murine SOD1 (cross-reaction) and no discernable staining in SOD1 knockout mice (Figure S6).

To further validate the finding of SOD1-staining inclusions in ALS, antibodies were raised in chicken against three of the SOD1 peptides. The antibodies showed high specificities for SOD1 in western blots (Figure S7). Immobilized on Sepharose they efficiently bound denatured but not native SOD1 (Figure S4). Histopathologically, they all stained granular inclusions in motoneurons of ALS cases in a fashion indistinguishable from that of the rabbit antibodies, and there were no immunopositive inclusions in controls (Figure S7).

### SOD1 inclusions are hallmarks of ALS patients carrying SOD1 mutations

Inclusions containing SOD1 are considered hallmarks of ALS caused by mutations in the enzyme [22], [28], [29]. Using the present anti-peptide antibodies, SOD1-immunoreactive inclusions have previously been demonstrated in patients carrying the G72C and A4V mutations [30], and in a case carrying the G127X C-terminal truncation mutation [9]. With the latter, the 4–20Ra-ab anti-SOD1 antibody allowed visualization of the inclusions, but not the 131–153Ra-ab anti-SOD1 antibody, which is directed against a sequence absent from the mutant protein. In most patients carrying SOD1 mutations, the inclusions tend to be larger and both skein- and Lewy body-like [9], [30], [31] (e.g. Figure 1H), but small granular SOD1-immunoreactive inclusions were also found in the patient with the G127X mutation (Figure 1I). Importantly, in patients homozygous for the wild type-like D90A mutant SOD1, the majority of the SOD1-immunoreactive inclusions were of the small granular type (Figure 1G).

### Biochemical analysis of SOD1 in ALS patients and controls

Homogenates of tissues from the temporal lobe, the precentral gyrus and from the cervical and lumbar spinal cord ventral horns of 5 SALS patients, 4 FALS patients and 5 control patients were examined by western blot using anti-SOD1 peptide antibodies. The denatured SOD1 monomer was seen as a single band of the appropriate molecular weight in all samples (Figure 3). There was no evidence of smearing or accumulation of SOD1 protein in the loading wells, nor were there any other alterations detected with the three antibodies used that could correlate with disease.

The activites of SOD isoenzymes were analysed in 15 different parts of the CNS from controls, SALS and FALS patients (Tables S1 and S2). SOD1 showed throughout the highest activity, and there were no significant differences in this enzyme between the groups. The SOD1 activities in the areas mainly afflicted by ALS, the spinal cord ventral horns and the precentral gyrus, were intermediate among gray matter areas. Using the specific activity of human SOD1 [32], the concentration of the enzyme was calculated to be 2 µM. Ventral horns were found to contain about 60 mg protein per g wet weight. SOD1 thus accounts for about 0.1% of the tissue protein.

## Discussion

In this report we describe a small round inclusion characterized by its immunoreactivity with anti-SOD1 antibodies and having a size of approximately 0.5–3 µm in SALS and non-SOD1 FALS patients. The inclusions occurred in large spinal cord neurons such as motoneurons and Clarke's column neurons. We found the inclusion in all 37 ALS patients we examined, as well as in the 2 SBMA patients, but only in 5 of 47 controls; and in those, to a much lesser extent. Inclusions containing SOD1 are considered to be hallmarks of ALS caused by mutations in the enzyme [9], [22], [28], [30]. Accordingly, using the present anti-SOD1 peptide antibodies on sections from such ALS patients, both larger skein- and Lewy-body like inclusions and the small granular inclusions were found.

The peptides used for antibody production cover more than 80% of the SOD1 sequence. Some of these sequence segments are hidden in native SOD1, whereas others are partially exposed on the protein surface. The longest continuous exposed stretches are found in the 24–39, 57–72 and 80–96 segments. Even so, all anti-peptide antibodies failed to recognize native SOD1, suggesting that the primary affinity is to epitopes in non-native configurations, i.e. to bind the antibody the peptide segments need to be flexible or distorted away from their native geometries. The anti-peptide antibodies showed uniform high affinities for denatured SOD1 *in vitro* (Figures 2 and 3; Figures S3, S4 and S7), and they all stained SOD1 in the inclusions (Figure 1; Figures S1, S2 and S7). This suggests that the SOD1 detected was in a non-native state, and either conformationally heterogeneous or disordered (allowing induced fits). An earlier study [33] using an antibody directed towards a peptide in the SOD1 dimer interface, however, failed to detect SOD1 inclusions in non-SOD1 FALS cases.

The 146 mutations in SOD1 so far associated with ALS probably cause disease by essentially the same mechanism [6]. The levels of mutant SOD1 in spinal ventral horns of patients carrying SOD1 mutations vary from equal [15] to around 1% of controls [9]. The lowest levels are found in the C-terminal truncation mutations [9]. These lack a β-strand in the β-barrel core of the subunits and the stabilizing C57-C147 disulfide bond and therefore cannot adopt native folding. Cytotoxic conformational species of SOD1 common to all ALS types are hence likely to be misfolded, and may exist in very low concentrations.

Consequently, the amounts of misfolded SOD1 in the granular inclusions are also likely to be minute and difficult to detect against the background native cytosolic SOD1. The present success is based on a combination of two factors: the use of anti-peptide antibodies specific for non-native/misfolded SOD1, and screening with a set of such antibodies covering the major part of the SOD1 sequence. Previous studies on ALS patients lacking SOD1 mutations have resulted in detection of occasional Lewy body-like structures staining for SOD1 [22], [27], [28], [29] as we also found, or failure to detect any inclusion staining [23], [25]. In these studies, antibodies raised against whole SOD1 were used, which might explain the different outcomes.

SOD1-containing inclusions, as well as detergent-resistant aggregates of SOD1, are also found in ALS animal models in which mutant SOD1s are transgenically overexpressed [9], [11], [12], [34], [35]. Whether the inclusions/aggregates or some precursor non-native/misfolded form of SOD1 is responsible for the toxicity is still a matter of debate [12], [36], [37]. Evidence of noxious effects in the spinal cord can be seen relatively early [38], [39], [40], [41], whereas the inclusions and aggregates accumulate in the terminal symptomatic phase of the disease [11], [12], [35]. The inclusions are perhaps terminal markers in cells compromised by long-term assault from cytotoxic SOD1 species.

By confocal microscopy we found no evidence for colocalization between the granular SOD1 inclusions and markers for the major somal compartments endoplasmic reticulum and mitochondria (Figure S1). This suggests that the SOD1 inclusions are mainly cytosolic. There was, however, a partial colocalization with cathepsin D, indicating that the inclusions become targeted for degradation via the lysosomal pathway (Figure 1O).

The selective vulnerability of motor areas to the toxic effects of mutant forms of the ubiquitously expressed SOD1 is a conundrum. Compared to other organs in the human body, the levels of SOD1 in the CNS are relatively low [42]. Here we have shown that SOD1 levels in motor areas are only intermediately high compared to other gray matter areas (Table S1). Overall, SOD1 is moderately expressed and we found no differences in amounts of SOD1 between ALS patients and controls.

To conclude, we have found that granular inclusions containing misfolded SOD1 as a rule exist in motoneurons of both sporadic and familial ALS patients lacking SOD1 mutations. Although the importance of SOD1 inclusions for the pathogenesis of ALS is unknown, the findings provide circumstantial evidence that the wild-type SOD1 protein may participate in the pathogenesis of ALS. The presence of the inclusions also in familial cases and in the two SBMA patients suggests that in motoneuron disease induced by mutations in other genes, SOD1 may still be involved in downstream events.

## Materials and Methods

### Antibodies

The anti-SOD1 antibodies used were polyclonal rabbit antibodies raised against keyhole limpet hemocyanin-coupled peptides corresponding to amino acids 4–20, 24–39, 43–57, 57–72, 80–96, 100–115 and 131–153 in the human SOD1 sequence or corresponding chicken antibodies raised against peptides corresponding to amino acids 24–37, 57–72 and 131–153. They were purified on a Sulfolink coupling gel with the corresponding peptide coupled [12]. An antibody against a neopeptide in G127X mutant SOD1 was similarily prepared [9]. The other antibodies used are presented in Table S3.

### Tests of the anti-SOD1 antibodies by ELISA

ELISA plates with 96 wells (Maxisorp, Nunc A/S, Roskilde, Denmark) were coated overnight at 4°C with 100 µl of 2 µg/ml native human SOD1 (Sigma, Schelldorf, Germany) or human SOD1 that had been denatured with 4 M guanidinium chloride and 5 mM of the chelator diethylenetriaminepentaacetic acid (DTPA). This resulted in a complete denaturation of the SOD1 (Figure S7). After washing and blocking, primary antibodies were added and incubated at 23°C for 90 min. Finally, peroxidase-labeled secondary antibodies were added followed by development in substrate (1,2-phenylenediamine and H_2_O_2_) for 6 min, and the absorbance was read at 490 nm. Complete reactions in wells without SOD1 coating were used as blanks and the ELISA readings were subtracted. For the peptide antibodies, the subtractions amounted to an A_490_ of 0.015–0.020. Secondary antibodies alone gave negligible reactions in wells coated with SOD1.

### Subjects

Tissues were collected at autopsy from patients prospectively enrolled at the Department of Neurology, Umeå University Hospital. All patients fulfilled the El Escorial criteria for ALS [43]. This group consisted of 16 patients with SALS (mean age 70±9 [49–83] years) and 6 patients with FALS (mean age 63±4 [55–68] years). All 22 ALS patients were genotyped for SOD1 mutations as described previously [4], and none were found.

Furthermore, a retrospective search for clinicopathologically confirmed cases of ALS from the hospital records yielded 2 FALS patients (aged 33 and 49 years) and 13 SALS patients (mean age 70±10 [55–88] years), from whom material was subjected to histological study. No statistically significant differences between prospectively and retrospectively collected patients with the same type of ALS were found, and the materials were combined in the final analysis, giving one group of 8 FALS patients (mean age 58±12 [33–68]; years) and one group of 29 SALS patients (mean age 70±9 [49–88] years). From the hospital records two cases with SBMA (aged 52 and 76 years, respectively) were found. The diagnosis was verified by the presence of the trinucleotide repeats in the androgen receptor. For comparison, spinal cord tissue from two patients with SOD1 gene mutations (D90A and G127insTGGG [G127X]), respectively, was also subjected to histological study.

Control tissue from 28 control patients with other neurodegenerative diseases (mean age 74±19 [2–92]; 15 with Alzheimer's disease; 7 with Parkinson's disease; 3 with multiple sclerosis; 1 with frontotemporal lobar degeneration; 1 with tuberous sclerosis; 1 with Huntington's disease) and from 19 control patients without neurological disease (mean age 69±17 [37–91] years; the patients died from heart conditions or pneumonia) were used. Of these, 7 patients with neurodegenerative diseases and 3 without neurodegenerative diseases were prospectively enrolled and the rest collected from archival material.

The study adhered to the tenets of the Helsinki Declaration. Information about the study was given orally and in written format to next of kin, and in most cases also to the patient. Informed consent was obtained from next of kin from all patients prospectively enrolled in the study, the oral consent was then noted in the hospital files. The procedure was approved by the Ethical Committee of Umeå University which also approved the use of retrospective archival material from the hospital laboratory of clinical pathology.

### Microscopy

For the prospectively collected material, tissue samples were taken from the cervical, thoracic and lumbar regions, and for the retrospective material the tissues available mostly consisted only of blocks from the cervical and/or thoracic parts of the spinal cord. Tissue samples were also taken from hippocampus, ventral cingulate gyrus and frontal cortex, middle and superior temporal gyrus, striatum and mesencephalon. Tissues for histopathological studies were immersion-fixed in 4% paraformaldehyde in 0.1 M Na phosphate, pH 7.4. The immunohistochemical and fluorescent microscopy procedures are described in detail in Supporting Information (Material and Methods).

### Western blot

Tissues were homogenized as described in Supporting Information (Material and Methods) and were without prior centrifugation diluted 1:1 in SDS-PAGE loading buffer, heated for 5 min at 95°C and separated on 12% Ready Gels (BioRad, Hercules, CA, USA). Probing and detection were done as previously described [42], [44].

### Statistics

Statistical analysis was done using the STATISTICA data analysis software system (version 7.1; StatSoft Inc., Tulsa, OK, USA) or SPSS (version 15; SPSS Inc., Chicago, IL, USA). The nonparametric Kruskall-Wallis statistics (when comparing three groups) and Mann-Whitney statistics (when comparing two groups) were used for the morphological comparisons. In estimation of staining, the numbers given are median and range. When comparing SOD activities, analysis of variance (ANOVA) was used.

## Supporting Information

Figure S1 Confocal micrographs of sections from the lumbar spinal cord of SALS patients. The sections were double-labeled with the 57-72Ra-ab anti-SOD1 peptide antibody (green in B, C, E, F, H, I, K and L) and antibodies against either the endoplasmic reticulum marker GRP 78 (red in A and C), the mitochondrial marker mitochondrial Hsp70 (mHSP70; red in D and F), TDP-43 (red in G and I) or ubiquitin (red in J and L). Micrographs of the green channel scan showing small granular SOD1-immunoreactive inclusions (C, F, I and K). Corresponding SOD1-immunoreactive inclusions have been marked by yellow arrowheads in K and L. Micrographs of the red channel showing skein-like inclusions (J and L). Merged pictures of green and red channel scan not showing any overlap of green and red fluorescence, and thus not detecting any localization of small granular SOD1-immunoreactive inclusions in the endoplasmic reticulum and mitochondria or in TDP-43 or ubiquitin-containing inclusions, respectively.). Scale bar  = 14 µm (in A-C), 9 µm (in D-F), 5 µm (in G-I), and 8 µm (in J-L).
(6.03 MB TIF)

Figure S2 Micrographs depicting SOD1- or TDP-43-immunoreactive inclusions in spinal cord motoneurons (A-C), the effect of preincubation of the primary antibody with the peptide used as immunogen (D-F), SOD1-immunohistochemistry of murine ventral horns (G-I), and absence of SOD1-immunoreactivity in a patient with frontotemporal lobar degeneration (J, K). Sections from a SBMA patient with abundant inclusions in lumbar spinal cord motoneurons were stained with the 4-20Ra-ab anti-SOD1 peptide antibody (0.64 µg/ml) (A) or the anti-TDP-43 antibody (2 µg/ml) (B). Section from a FALS patient with abundant inclusions in lumbar spinal cord motoneurons. The section was stained with the anti-TDP-43 antibody (2 µg/ml) (C). In B and C typical skein-like inclusions are seen. Sections from a SALS patient with abundant inclusions in lumbar spinal cord motoneurons (D-F). The sections were stained with the 4-20Ra-ab anti-SOD1 peptide antibody (0.64 µg/ml). Several small granular inclusions in the soma was seen when the antibody was preincubated only with diluent (D). The small granular inclusions were only weakly detectable when the antibody was preincubated with an intermediate concentration of the immunizing peptide (1.4 µg/ml) (E). No SOD1-positive structures were detected when the antibody was preincubated with a high concentration of the immunizing peptide (0.14 mg/ml) (F). Sections of murine lumbar ventral horns stained with the 4-20Ra-ab anti-SOD1 peptide antibody (0.64 µg/ml) (G-I). The mouse transgenically overexpressing G93A mutant human SOD1 showed abundant staining for SOD1 (G). No staining for SOD1 was seen in the C57/Bl6 control mouse (H) or the SOD1 knock-out mouse (I). Sections from a patient with frontotemporal lobar degeneration showing no inclusions in either in lumbar spinal cord motoneurons (J) or in the anterior cingulate gyrus of the frontal lobe (K). The sections were stained with the 4-20Ra-ab anti-SOD1 peptide antibody (0.64 µg/ml). Confocal micrographs of sections from the lumbar spinal cord of SALS patients. The sections were double-labeled with the 57-72Chi-ab anti-SOD1 peptide antibody (green in L, N) and an antibody against autophagosome marker MAP1LC3A (red in M, N). Note absence of staining of MAP1LC3A. The scale bar  = 30 µm (in A-J), 90 µm (in K) and 20 µm (in L-N).
(8.65 MB TIF)

Figure S3Relative reactivities of antibodies towards native and denatured SOD1. ELISA plates were coated with either native (filled symbols in green) or denatured (unfilled symbols in red) SOD1, and were reacted with antibodies raised against peptides as described under Material and Methods. Threefold dilutions were made from a high antibody concentration giving an A490 of 1.0–1.7 with denatured SOD1. Reactivity of the 24–39Ra-ab (native SOD1  =  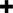, denatured SOD1  =  “), the 43–57Ra-ab (native  =  ▴, denatured  =  ▵), the 80–96Ra-ab (native SOD1  =  ▾, denatured SOD1 =  ▿), the 100–115Ra-ab (native SOD1  =  ▪, denatured SOD1  =  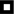) anti-SOD1 peptide antibodies. The highest concentrations were 0.1, 0.03, 0.03 and 0.03 µg/ml, respectively. The data presented are means of 4 wells for each point.(0.12 MB TIF)

Figure S4 Immunocapture of native and denatured SOD1 with immobilized antibodies. Antibodies immobilized on Sepharose were incubated for 1 h in pH 7.0 PBS containing 5 mg/ml of SOD1 that was either native (A, B) or had been denatured by exposure to guanidinium chloride and a chelator followed by dialysis (A, C). Following washes the bound SOD1 was analysed with western immunoblots. The amount of SOD1 in the incubations was in all cases more than 10-fold the maximal binding capacities of the antibodies. The native SOD1 solutions were incubated twice with the immobilized antibodies, with the intention to capture any traces of denatured SOD1 in the preparation with the first, to make the second more representative for the reaction (of the antipeptide antibodies) with native SOD1 (A, B). Note that among the antipeptide antibodies, only the rabbit (Ra-ab) and chicken (Ch-ab) 131-153 antibodies bound detectable amounts of native SOD1 (A, B). The amounts were in both cases more than 1000-fold lower than the amounts of denatured SOD1 bound. The C-terminal end is the part that folds last in SOD1 (Nordlund A, Oliveberg M (2006) Proc Natl Acad Sci U S A 103: 10218-10223) and the binding might be explained by partial unfolding caused by thermal fluctuations. The chicken (Ch-ab), sheep (Sh-ab; Calbiochem) and rabbit-1 (Ra-ab) antibodies versus whole SOD1 captured equally large amounts of native SOD1 in the two sequential incubations (B) as analyzed by a CCD-camera (ChemiDoc XRS, BioRad Inc.), data not shown.
(0.82 MB TIF)

Figure S5 Analysis of denatured and native SOD1 by hydrophobic interaction chromatography. Denatured SOD1 was prepared for the ELISA and immunocapture experiments by exposure to 4 M guanidinium chloride and 5 mM of the chelator DTPA followed by dialysis with PBS containing 1 mM DTPA. 250 µl of native or denatured SOD1 dissolved in PBS pH 7.0 at around 2 µg/ml were applied to 1 ml Octyl-Sepharose CL-4B (GE Biosciences) in columns. After 5 min, non-bound SOD1 was eluted with 2.5 ml of the PBS. Following washing with 10 ml PBS, SOD1 bound to the Octyl-Sepharose was eluted in 2.5 ml of PBS containing 4% SDS [8]. The initial SOD1 solutions (total), together with the non-bound and bound fractions were analysed by western immunoblotting using the 23-39Ra-ab anti-SOD1 peptide antibody. Native SOD1 is very hydrophilic and does not bind to the column [8]. Denatured SOD1 exposes hydrophobic internal structures, and the preparation used for the ELISA and immunocapture experiments (Figure 2, Figure S2 and S3), was found to bind quantitatively to the column.
(0.61 MB TIF)

Figure S6 Comparison of reactivities of antipeptide antibodies versus SOD1 in human and murine spinal cords. Equal amounts of extracts from ventral horns from a SALS and a FALS case and spinal cords from a SOD1 knockout mouse, a C57Bl6 control mouse and a wild-type human SOD1 transgenic mouse were examined by western blots using the 4–Ra-ab, 57–Ra-ab, 131–Ra-ab and 131–Ch-ab antibodies. In none of the cases was any reaction seen with proteins in the knockout homogenate, demonstrating the specificity of the antibodies. The 131–153 sequence is equal in human and mouse SOD1 and similar reactions are found in the extracts from the ALS cases and the control mouse. The other two antibodies showed lower cross-reactivities with murine SOD1 as expected.
(1.80 MB TIF)

Figure S7 Confocal micrographs of sections from ventral horns of SALS patients and neurological control patients, and analysis of SOD1 by western blot. The micrographs depict the findings of the three different chicken antibodies (24-37Ch-ab, 58-72Ch-ab, 131-153Ch-ab, respectively; green fluorescence). In the SALS patients small granular inclusions were seen with all three antibodies. In the neurodegenerative and non-neurological control patients no inclusions were seen in motoneurons. Homogenates of tissue from the spinal cord ventral horns from one control patient, one SALS and one FALS patients were analyzed by western blots, using the 24-37Ch-ab, 57-72Ch-ab and 131-153Ch-ab anti-SOD1 peptide antibodies. Short, intermediate and long exposures are presented. Weak nonspecific bands at about 51, 41 and 32 kDa were seen with the 24-37Ch-ab anti-SOD1 antibody. Since they were not seen with the other antibodies they are probably not related to SOD1 and thus unspecific. The total intensities were estimated at approximately 3% of that of the SOD1 monomer. The scale bars are 18 µm.
(4.17 MB TIF)

Table S1 SOD1 activities in different CNS areas from controls and ALS patients.
(0.04 MB DOC)

Table S2 SOD2 and SOD3 activities in different CNS areas from controls, SALS and FALS patients.
(0.05 MB DOC)

Table S3 Antibodies used in morphological studies.
(0.05 MB DOC)

## References

[1] Haverkamp LJ, Appel V, Appel SH (1995). Natural history of amyotrophic lateral sclerosis in a database population. Validation of a scoring system and a model for survival prediction.. Brain.

[2] Rosen DR, Siddique T, Patterson D, Figlewicz DA, Sapp P (1993). Mutations in Cu/Zn superoxide dismutase gene are associated with familial amyotrophic lateral sclerosis.. Nature.

[3] Gurney ME, Pu H, Chiu AY, Dal Canto MC, Polchow CY (1994). Motor neuron degeneration in mice that express a human Cu,Zn superoxide dismutase mutation.. Science.

[4] Andersen PM, Nilsson P, Ala-Hurula V, Keranen ML, Tarvainen I (1995). Amyotrophic lateral sclerosis associated with homozygosity for an Asp90Ala mutation in CuZn-superoxide dismutase.. Nat Genet.

[5] Reaume AG, Elliott JL, Hoffman EK, Kowall NW, Ferrante RJ (1996). Motor neurons in Cu/Zn superoxide dismutase-deficient mice develop normally but exhibit enhanced cell death after axonal injury.. Nat Genet.

[6] Andersen PM, Sims KB, Xin WW, Kiely R, O'Neill G (2003). Sixteen novel mutations in the Cu/Zn superoxide dismutase gene in amyotrophic lateral sclerosis: a decade of discoveries, defects and disputes.. Amyotroph Lateral Scler Other Motor Neuron Disord.

[7] Forman MS, Trojanowski JQ, Lee VM (2004). Neurodegenerative diseases: a decade of discoveries paves the way for therapeutic breakthroughs.. Nat Med.

[8] Valentine JS, Hart PJ (2003). Misfolded CuZnSOD and amyotrophic lateral sclerosis.. Proc Natl Acad Sci U S A.

[9] Jonsson PA, Ernhill K, Andersen PM, Bergemalm D, Brannstrom T (2004). Minute quantities of misfolded mutant superoxide dismutase-1 cause amyotrophic lateral sclerosis.. Brain.

[10] Lindberg MJ, Bystrom R, Boknas N, Andersen PM, Oliveberg M (2005). Systematically perturbed folding patterns of amyotrophic lateral sclerosis (ALS)-associated SOD1 mutants.. Proc Natl Acad Sci U S A.

[11] Wang J, Xu G, Slunt HH, Gonzales V, Coonfield M (2005). Coincident thresholds of mutant protein for paralytic disease and protein aggregation caused by restrictively expressed superoxide dismutase cDNA.. Neurobiol Dis.

[12] Jonsson PA, Graffmo KS, Andersen PM, Brannstrom T, Lindberg M (2006). Disulphide-reduced superoxide dismutase-1 in CNS of transgenic amyotrophic lateral sclerosis models.. Brain.

[13] Bowling AC, Barkowski EE, McKenna-Yasek D, Sapp P, Horvitz HR (1995). Superoxide dismutase concentration and activity in familial amyotrophic lateral sclerosis.. J Neurochem.

[14] Sato T, Nakanishi T, Yamamoto Y, Andersen PM, Ogawa Y (2005). Rapid disease progression correlates with instability of mutant SOD1 in familial ALS.. Neurology.

[15] Jonsson PA, Graffmo KS, Andersen PM, Marklund SL, Brannstrom T (2009). Superoxide dismutase in amyotrophic lateral sclerosis patients homozygous for the D90A mutation.. Neurobiol Dis.

[16] Jonsson PA, Graffmo KS, Brannstrom T, Nilsson P, Andersen PM (2006). Motor neuron disease in mice expressing the wild type-like D90A mutant superoxide dismutase-1.. J Neuropathol Exp Neurol.

[17] Jaarsma D, Haasdijk ED, Grashorn JA, Hawkins R, van Duijn W (2000). Human Cu/Zn superoxide dismutase (SOD1) overexpression in mice causes mitochondrial vacuolization, axonal degeneration, and premature motoneuron death and accelerates motoneuron disease in mice expressing a familial amyotrophic lateral sclerosis mutant SOD1.. Neurobiol Dis.

[18] Deng HX, Shi Y, Furukawa Y, Zhai H, Fu R (2006). Conversion to the amyotrophic lateral sclerosis phenotype is associated with intermolecular linked insoluble aggregates of SOD1 in mitochondria.. Proc Natl Acad Sci U S A.

[19] Rakhit R, Crow JP, Lepock JR, Kondejewski LH, Cashman NR (2004). Monomeric Cu,Zn-superoxide dismutase is a common misfolding intermediate in the oxidation models of sporadic and familial amyotrophic lateral sclerosis.. J Biol Chem.

[20] Ezzi SA, Urushitani M, Julien JP (2007). Wild-type superoxide dismutase acquires binding and toxic properties of ALS-linked mutant forms through oxidation.. J Neurochem.

[21] Gruzman A, Wood WL, Alpert E, Prasad MD, Miller RG (2007). Common molecular signature in SOD1 for both sporadic and familial amyotrophic lateral sclerosis.. Proc Natl Acad Sci U S A.

[22] Shibata N, Hirano A, Kobayashi M, Sasaki S, Kato T (1994). Cu/Zn superoxide dismutase-like immunoreactivity in Lewy body-like inclusions of sporadic amyotrophic lateral sclerosis.. Neurosci Lett.

[23] Ince PG, Shaw PJ, Slade JY, Jones C, Hudgson P (1996). Familial amyotrophic lateral sclerosis with a mutation in exon 4 of the Cu/Zn superoxide dismutase gene: pathological and immunocytochemical changes.. Acta Neuropathol (Berl).

[24] Shaw CE, Enayat ZE, Powell JF, Anderson VE, Radunovic A (1997). Familial amyotrophic lateral sclerosis. Molecular pathology of a patient with a SOD1 mutation.. Neurology.

[25] Shaw PJ, Chinnery RM, Thagesen H, Borthwick GM, Ince PG (1997). Immunocytochemical study of the distribution of the free radical scavenging enzymes Cu/Zn superoxide dismutase (SOD1); MN superoxide dismutase (MN SOD) and catalase in the normal human spinal cord and in motor neuron disease.. J Neurol Sci.

[26] Ince PG, Tomkins J, Slade JY, Thatcher NM, Shaw PJ (1998). Amyotrophic lateral sclerosis associated with genetic abnormalities in the gene encoding Cu/Zn superoxide dismutase: molecular pathology of five new cases, and comparison with previous reports and 73 sporadic cases of ALS.. J Neuropathol Exp Neurol.

[27] Liu Y, Brooks BR, Taniguchi N, Hartmann HA (1998). CuZnSOD and MnSOD immunoreactivity in brain stem motor neurons from amyotrophic lateral sclerosis patients.. Acta Neuropathol (Berl).

[28] Kato S, Takikawa M, Nakashima K, Hirano A, Cleveland DW (2000). New consensus research on neuropathological aspects of familial amyotrophic lateral sclerosis with superoxide dismutase 1 (SOD1) gene mutations: inclusions containing SOD1 in neurons and astrocytes.. Amyotroph Lateral Scler Other Motor Neuron Disord.

[29] Hirano A (1998). Neuropathology of familial amyotrophic lateral sclerosis patients with superoxide dismutase 1 gene mutations.. Neuropathology.

[30] Stewart HG, Mackenzie IR, Eisen A, Brannstrom T, Marklund SL (2006). Clinicopathological phenotype of ALS with a novel G72C SOD1 gene mutation mimicking a myopathy.. Muscle Nerve.

[31] Shibata N, Hirano A, Kobayashi M, Siddique T, Deng HX (1996). Intense superoxide dismutase-1 immunoreactivity in intracytoplasmic hyaline inclusions of familial amyotrophic lateral sclerosis with posterior column involvement.. J Neuropathol Exp Neurol.

[32] Marklund SL, Andersen PM, Forsgren L, Nilsson P, Ohlsson PI (1997). Normal binding and reactivity of copper in mutant superoxide dismutase isolated from amyotrophic lateral sclerosis patients.. J Neurochem.

[33] Liu HN, Sanelli T, Horne P, Pioro EP, Strong MJ (2009). Lack of evidence of monomer/misfolded superoxide dismutase-1 in sporadic amyotrophic lateral sclerosis.. Ann Neurol.

[34] Bruijn LI, Houseweart MK, Kato S, Anderson KL, Anderson SD (1998). Aggregation and motor neuron toxicity of an ALS-linked SOD1 mutant independent from wild-type SOD1.. Science.

[35] Wang J, Xu G, Gonzales V, Coonfield M, Fromholt D (2002). Fibrillar inclusions and motor neuron degeneration in transgenic mice expressing superoxide dismutase 1 with a disrupted copper-binding site.. Neurobiol Dis.

[36] Bruijn LI, Miller TM, Cleveland DW (2004). Unraveling the mechanisms involved in motor neuron degeneration in ALS.. Annu Rev Neurosci.

[37] Zetterstrom P, Stewart HG, Bergemalm D, Jonsson PA, Graffmo KS (2007). Soluble misfolded subfractions of mutant superoxide dismutase-1s are enriched in spinal cords throughout life in murine ALS models.. Proc Natl Acad Sci U S A.

[38] Williamson TL, Cleveland DW (1999). Slowing of axonal transport is a very early event in the toxicity of ALS-linked SOD1 mutants to motor neurons.. Nat Neurosci.

[39] Pasinelli P, Houseweart MK, Brown RH, Cleveland DW (2000). Caspase-1 and -3 are sequentially activated in motor neuron death in Cu,Zn superoxide dismutase-mediated familial amyotrophic lateral sclerosis.. Proc Natl Acad Sci U S A.

[40] Feeney SJ, McKelvie PA, Austin L, Jean-Francois MJ, Kapsa R (2001). Presymptomatic motor neuron loss and reactive astrocytosis in the SOD1 mouse model of amyotrophic lateral sclerosis.. Muscle Nerve.

[41] Wooley CM, Sher RB, Kale A, Frankel WN, Cox GA (2005). Gait analysis detects early changes in transgenic SOD1(G93A) mice.. Muscle Nerve.

[42] Marklund SL (1984). Extracellular superoxide dismutase in human tissues and human cell lines.. J Clin Invest.

[43] (1994). Papers from the 3rd International Symposium on Amyotrophic Lateral Sclerosis/Motor Neurone Disease. Genetics and Cell Biology of the Motor Neurone. Birmingham, United Kingdom, November 2-4, 1992.. J Neurol Sci.

[44] Jacobsson J, Jonsson PA, Andersen PM, Forsgren L, Marklund SL (2001). Superoxide dismutase in CSF from amyotrophic lateral sclerosis patients with and without CuZn-superoxide dismutase mutations.. Brain.

