# Supplementary figures and images for: Novel Antibodies Reveal Inclusions Containing Non-Native SOD1 in Sporadic ALS Patients
Source: PLoS One. 2010 Jul 14;5(7):e11552. doi: 10.1371/journal.pone.0011552 (PMC2904380; doi:10.1371/journal.pone.0011552)

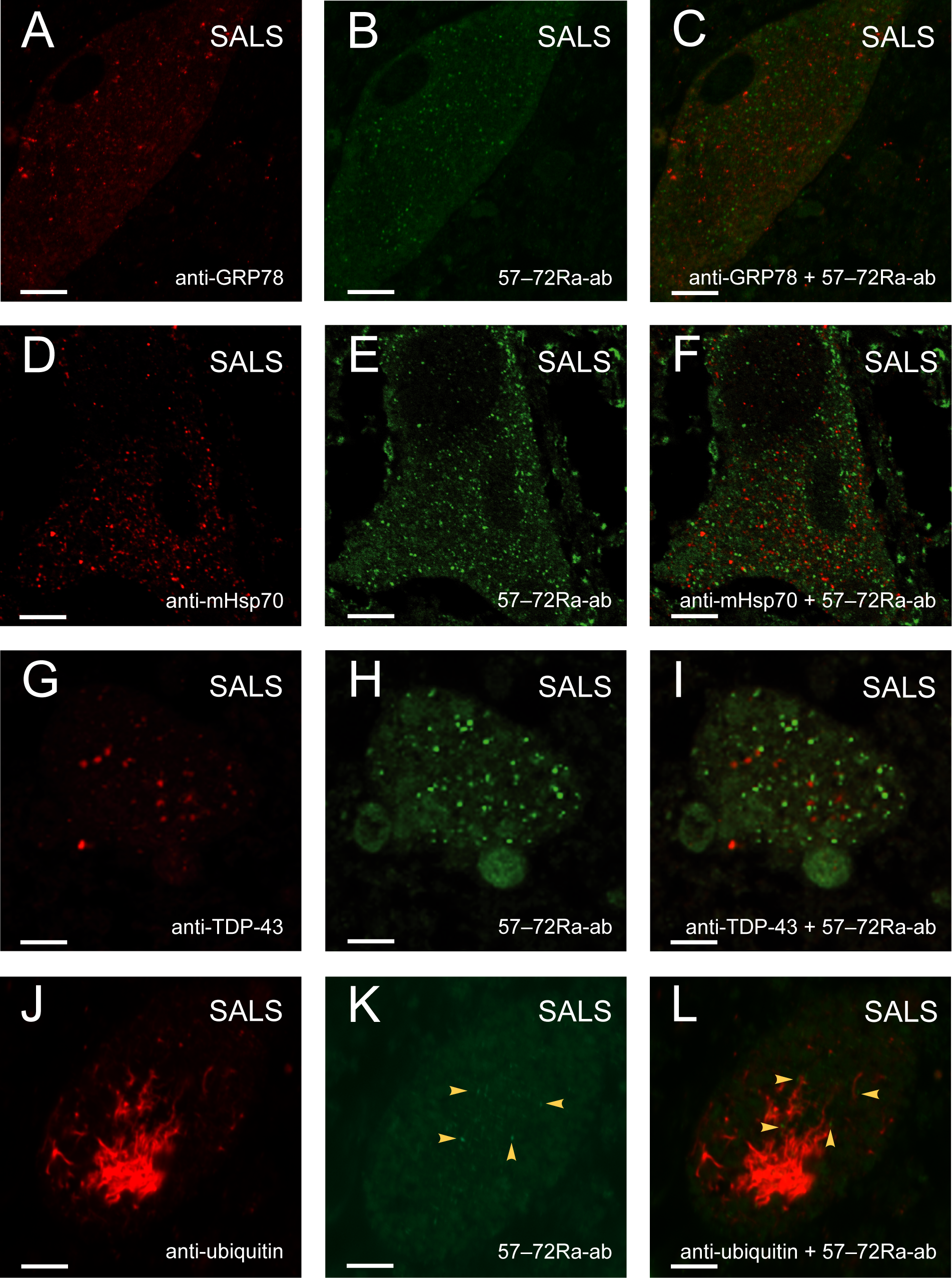

Supplement: Figure S1 — Confocal micrographs of sections from the lumbar spinal cord of SALS patients. The sections were double-labeled with the 57-72Ra-ab anti-SOD1 peptide antibody (green in B, C, E, F, H, I, K and L) and antibodies against either the endoplasmic reticulum marker GRP 78 (red in A and C), the mitochondrial marker mitochondrial Hsp70 (mHSP70; red in D and F), TDP-43 (red in G and I) or ubiquitin (red in J and L). Micrographs of the green channel scan showing small granular SOD1-immunoreactive inclusions (C, F, I and K). Corresponding SOD1-immunoreactive inclusions have been marked by yellow arrowheads in K and L. Micrographs of the red channel showing skein-like inclusions (J and L). Merged pictures of green and red channel scan not showing any overlap of green and red fluorescence, and thus not detecting any localization of small granular SOD1-immunoreactive inclusions in the endoplasmic reticulum and mitochondria or in TDP-43 or ubiquitin-containing inclusions, respectively.). Scale bar = 14 µm (in A-C), 9 µm (in D-F), 5 µm (in G-I), and 8 µm (in J-L). (6.03 MB TIF) [file pone.0011552.s001.tif]

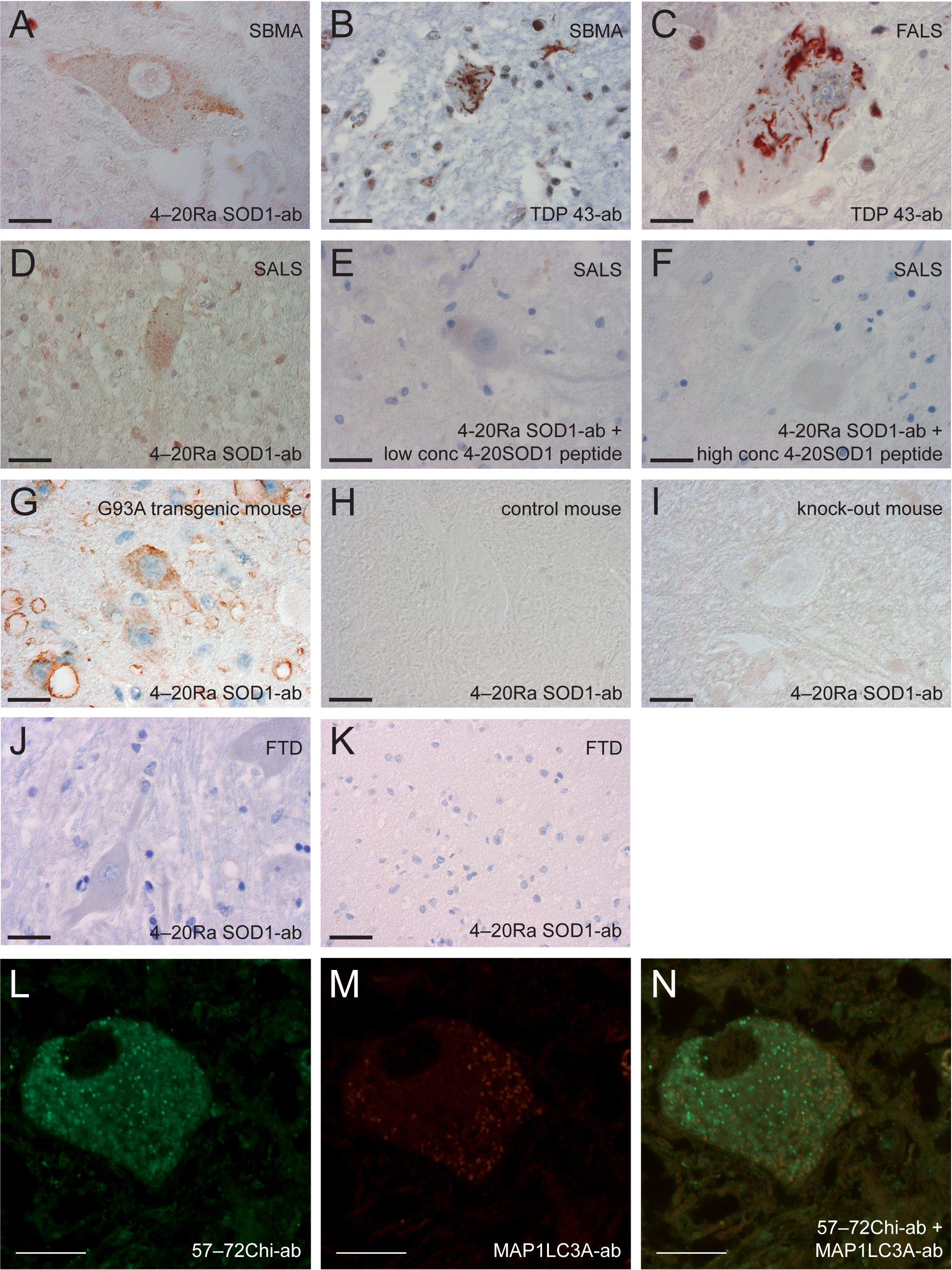

Supplement: Figure S2 — Micrographs depicting SOD1- or TDP-43-immunoreactive inclusions in spinal cord motoneurons (A-C), the effect of preincubation of the primary antibody with the peptide used as immunogen (D-F), SOD1-immunohistochemistry of murine ventral horns (G-I), and absence of SOD1-immunoreactivity in a patient with frontotemporal lobar degeneration (J, K). Sections from a SBMA patient with abundant inclusions in lumbar spinal cord motoneurons were stained with the 4-20Ra-ab anti-SOD1 peptide antibody (0.64 µg/ml) (A) or the anti-TDP-43 antibody (2 µg/ml) (B). Section from a FALS patient with abundant inclusions in lumbar spinal cord motoneurons. The section was stained with the anti-TDP-43 antibody (2 µg/ml) (C). In B and C typical skein-like inclusions are seen. Sections from a SALS patient with abundant inclusions in lumbar spinal cord motoneurons (D-F). The sections were stained with the 4-20Ra-ab anti-SOD1 peptide antibody (0.64 µg/ml). Several small granular inclusions in the soma was seen when the antibody was preincubated only with diluent (D). The small granular inclusions were only weakly detectable when the antibody was preincubated with an intermediate concentration of the immunizing peptide (1.4 µg/ml) (E). No SOD1-positive structures were detected when the antibody was preincubated with a high concentration of the immunizing peptide (0.14 mg/ml) (F). Sections of murine lumbar ventral horns stained with the 4-20Ra-ab anti-SOD1 peptide antibody (0.64 µg/ml) (G-I). The mouse transgenically overexpressing G93A mutant human SOD1 showed abundant staining for SOD1 (G). No staining for SOD1 was seen in the C57/Bl6 control mouse (H) or the SOD1 knock-out mouse (I). Sections from a patient with frontotemporal lobar degeneration showing no inclusions in either in lumbar spinal cord motoneurons (J) or in the anterior cingulate gyrus of the frontal lobe (K). The sections were stained with the 4-20Ra-ab anti-SOD1 peptide antibody (0.64 µg/ml). Confocal micrographs of sections fro [file pone.0011552.s002.tif]

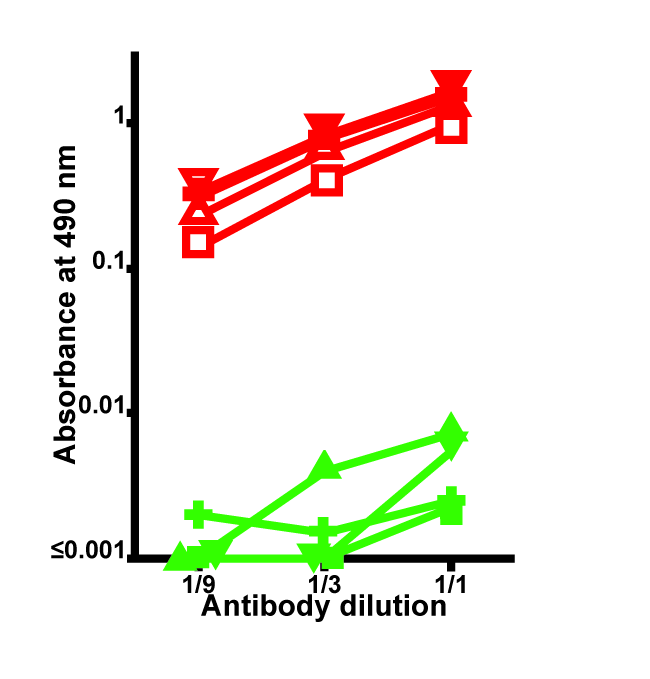

Supplement: Figure S3 — Relative reactivities of antibodies towards native and denatured SOD1. ELISA plates were coated with either native (filled symbols in green) or denatured (unfilled symbols in red) SOD1, and were reacted with antibodies raised against peptides as described under Material and Methods. Threefold dilutions were made from a high antibody concentration giving an A490 of 1.0–1.7 with denatured SOD1. Reactivity of the 24–39Ra-ab (native SOD1 = , denatured SOD1 = “), the 43–57Ra-ab (native = ▴, denatured = ▵), the 80–96Ra-ab (native SOD1 = ▾, denatured SOD1 = ▿), the 100–115Ra-ab (native SOD1 = ▪, denatured SOD1 = ) anti-SOD1 peptide antibodies. The highest concentrations were 0.1, 0.03, 0.03 and 0.03 µg/ml, respectively. The data presented are means of 4 wells for each point. (0.12 MB TIF) [file pone.0011552.s003.tif]

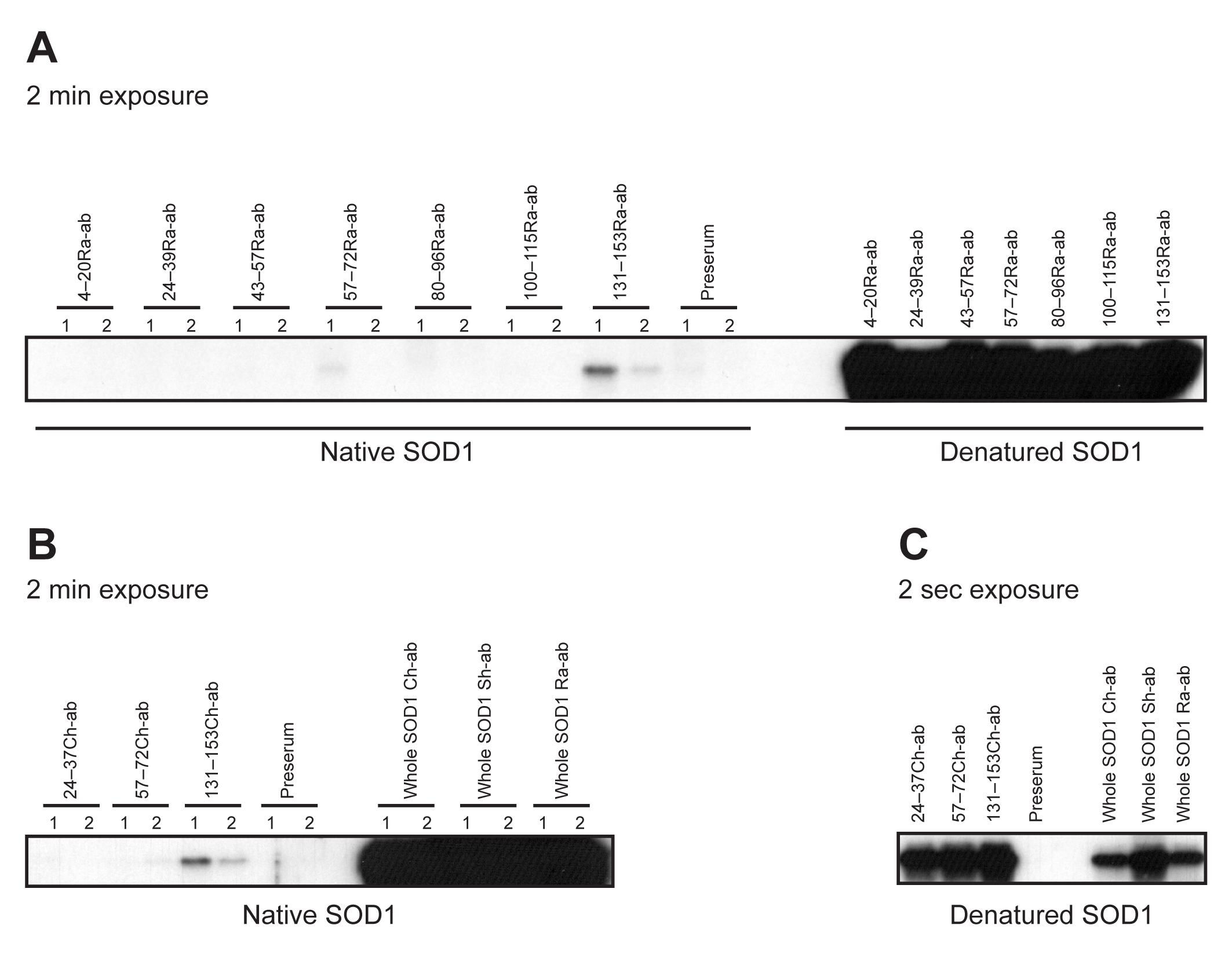

Supplement: Figure S4 — Immunocapture of native and denatured SOD1 with immobilized antibodies. Antibodies immobilized on Sepharose were incubated for 1 h in pH 7.0 PBS containing 5 mg/ml of SOD1 that was either native (A, B) or had been denatured by exposure to guanidinium chloride and a chelator followed by dialysis (A, C). Following washes the bound SOD1 was analysed with western immunoblots. The amount of SOD1 in the incubations was in all cases more than 10-fold the maximal binding capacities of the antibodies. The native SOD1 solutions were incubated twice with the immobilized antibodies, with the intention to capture any traces of denatured SOD1 in the preparation with the first, to make the second more representative for the reaction (of the antipeptide antibodies) with native SOD1 (A, B). Note that among the antipeptide antibodies, only the rabbit (Ra-ab) and chicken (Ch-ab) 131-153 antibodies bound detectable amounts of native SOD1 (A, B). The amounts were in both cases more than 1000-fold lower than the amounts of denatured SOD1 bound. The C-terminal end is the part that folds last in SOD1 (Nordlund A, Oliveberg M (2006) Proc Natl Acad Sci U S A 103: 10218-10223) and the binding might be explained by partial unfolding caused by thermal fluctuations. The chicken (Ch-ab), sheep (Sh-ab; Calbiochem) and rabbit-1 (Ra-ab) antibodies versus whole SOD1 captured equally large amounts of native SOD1 in the two sequential incubations (B) as analyzed by a CCD-camera (ChemiDoc XRS, BioRad Inc.), data not shown. (0.82 MB TIF) [file pone.0011552.s004.tif]

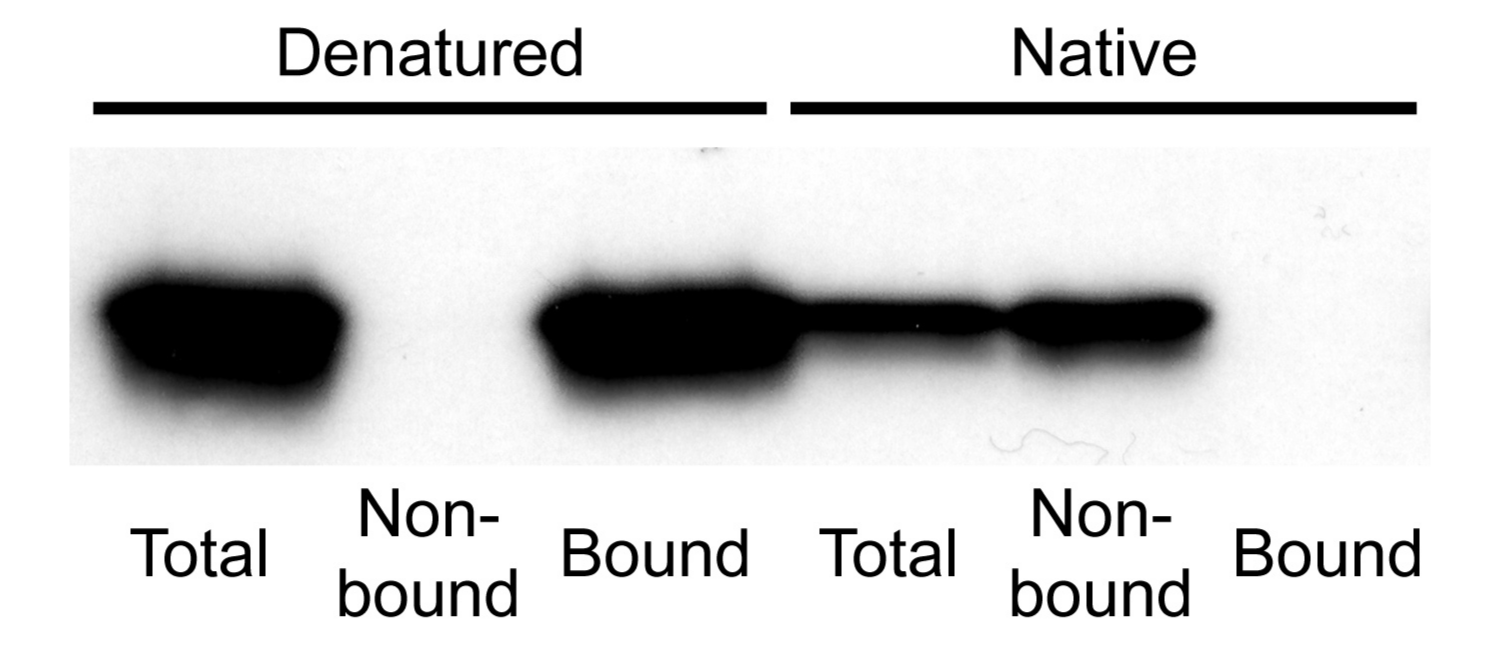

Supplement: Figure S5 — Analysis of denatured and native SOD1 by hydrophobic interaction chromatography. Denatured SOD1 was prepared for the ELISA and immunocapture experiments by exposure to 4 M guanidinium chloride and 5 mM of the chelator DTPA followed by dialysis with PBS containing 1 mM DTPA. 250 µl of native or denatured SOD1 dissolved in PBS pH 7.0 at around 2 µg/ml were applied to 1 ml Octyl-Sepharose CL-4B (GE Biosciences) in columns. After 5 min, non-bound SOD1 was eluted with 2.5 ml of the PBS. Following washing with 10 ml PBS, SOD1 bound to the Octyl-Sepharose was eluted in 2.5 ml of PBS containing 4% SDS [8]. The initial SOD1 solutions (total), together with the non-bound and bound fractions were analysed by western immunoblotting using the 23-39Ra-ab anti-SOD1 peptide antibody. Native SOD1 is very hydrophilic and does not bind to the column [8]. Denatured SOD1 exposes hydrophobic internal structures, and the preparation used for the ELISA and immunocapture experiments (Figure 2, Figure S2 and S3), was found to bind quantitatively to the column. (0.61 MB TIF) [file pone.0011552.s005.tif]

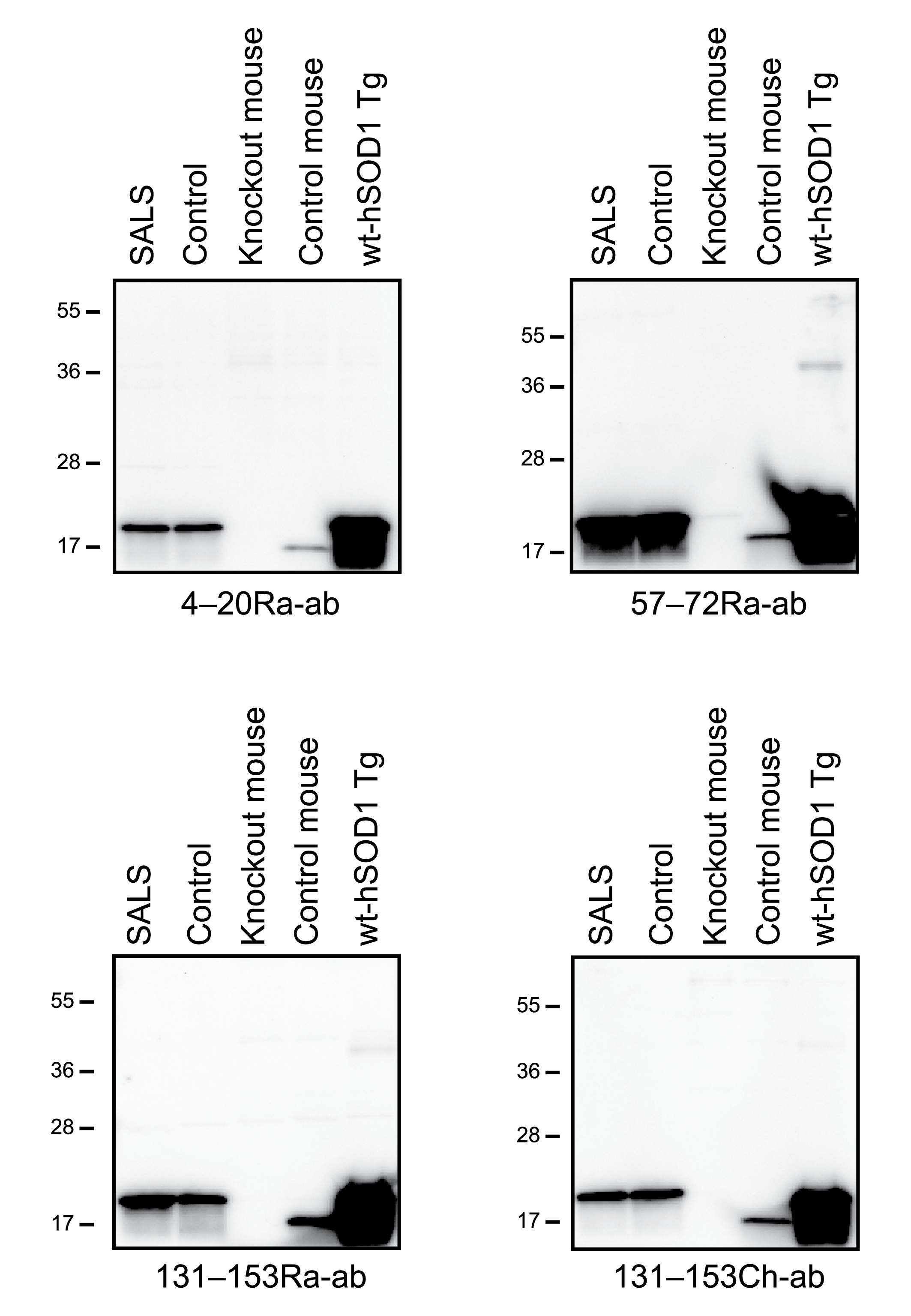

Supplement: Figure S6 — Comparison of reactivities of antipeptide antibodies versus SOD1 in human and murine spinal cords. Equal amounts of extracts from ventral horns from a SALS and a FALS case and spinal cords from a SOD1 knockout mouse, a C57Bl6 control mouse and a wild-type human SOD1 transgenic mouse were examined by western blots using the 4–Ra-ab, 57–Ra-ab, 131–Ra-ab and 131–Ch-ab antibodies. In none of the cases was any reaction seen with proteins in the knockout homogenate, demonstrating the specificity of the antibodies. The 131–153 sequence is equal in human and mouse SOD1 and similar reactions are found in the extracts from the ALS cases and the control mouse. The other two antibodies showed lower cross-reactivities with murine SOD1 as expected. (1.80 MB TIF) [file pone.0011552.s006.tif]

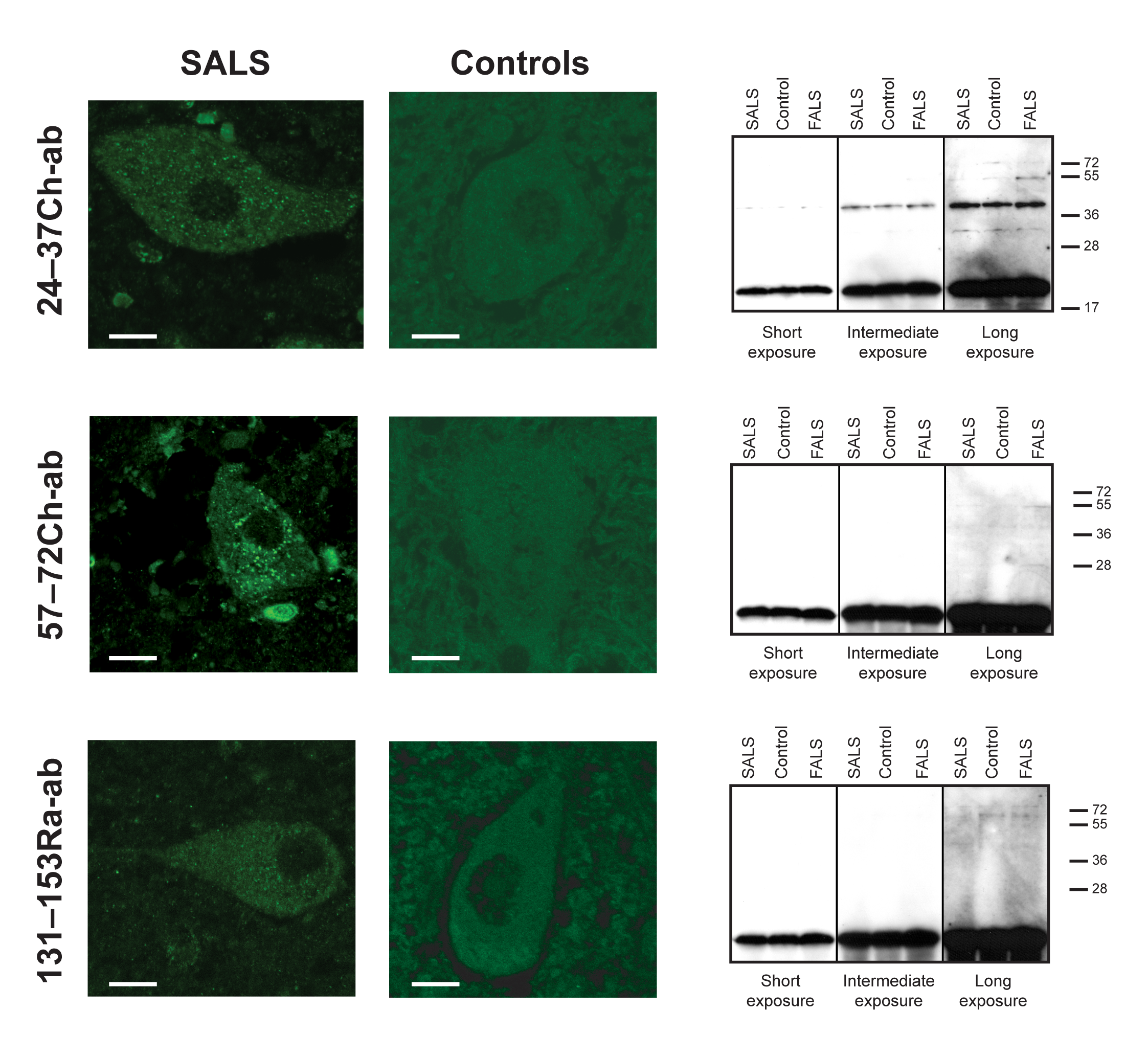

Supplement: Figure S7 — Confocal micrographs of sections from ventral horns of SALS patients and neurological control patients, and analysis of SOD1 by western blot. The micrographs depict the findings of the three different chicken antibodies (24-37Ch-ab, 58-72Ch-ab, 131-153Ch-ab, respectively; green fluorescence). In the SALS patients small granular inclusions were seen with all three antibodies. In the neurodegenerative and non-neurological control patients no inclusions were seen in motoneurons. Homogenates of tissue from the spinal cord ventral horns from one control patient, one SALS and one FALS patients were analyzed by western blots, using the 24-37Ch-ab, 57-72Ch-ab and 131-153Ch-ab anti-SOD1 peptide antibodies. Short, intermediate and long exposures are presented. Weak nonspecific bands at about 51, 41 and 32 kDa were seen with the 24-37Ch-ab anti-SOD1 antibody. Since they were not seen with the other antibodies they are probably not related to SOD1 and thus unspecific. The total intensities were estimated at approximately 3% of that of the SOD1 monomer. The scale bars are 18 µm. (4.17 MB TIF) [file pone.0011552.s007.tif]
