# Supplementary material for: Novel Antibodies Reveal Inclusions Containing Non-Native SOD1 in Sporadic ALS Patients
Source: PLoS One. 2010 Jul 14;5(7):e11552. doi: 10.1371/journal.pone.0011552 (PMC2904380; doi:10.1371/journal.pone.0011552)
Supplement: Table S1 — SOD1 activities in different CNS areas from controls and ALS patients. (0.04 MB DOC) [file pone.0011552.s008.doc]

| **Table S1.** SOD1 activities in different CNS areas from controls and ALS patients. | | | |
| --- | --- | --- | --- |
| **CNS area** | **Controls (n=5)** | **SALS (n=5)** | **FALS (n=4)** |
| Frontal lobe | 16,000 ± 2,300 | 16,200 ± 2,800 | 14,800 ± 2,800 |
| Temporal lobe | 15,100 ± 2,900 | 15,400 ± 1,400 | 15,400 ± 3,400 |
| Precentral gyrus | 14,600 ± 1,300 | 14,800 ± 800 | 16,800 ± 7,500 |
| Corona radiata | 12,900 ± 1,400 | 11,700 ± 1,200 | 13,200 ± 4,700 |
| Ventral vermis | 13,200 ± 3,000 | 16,000 ± 2,600 | 16,200 ± 4,100 |
| Lamina tecti | 17,000 ± 4,600 | 18,300 ± 2,300 | 17,200 ± 4,500 |
| Motor nuclei | 17,100 ± 1,300 | 17,600 ± 3,600 | 14,900 ± 1,500 |
| Cervical ventral horn | 11,900 ± 1,500 | 12,700 ± 2,800 | 11,300 ± 400 |
| Cervical dorsal funicle | 10,200 ± 800 | 12,600 ± 2,400 | 10,700 ± 5,900 |
| Cervical cortico-spinal tract | 9,200 ± 900 | 13,100 ± 3,400 | 11,900 ± 6,000 |
| Lumbar ventral horn | 13,800 ± 1,800 | 14,500 ± 1,400 | 15,400 ± 4,000 |
| Lumbar dorsal horn | 13,600 ± 1,700 | 12,500 ± 2,000 | 14,500 ± 3,200 |
| Lumbar dorsal funicle | 11,700 ± 1,100 | 11,600 ± 1,000 | 10,700 ± 1000 |
| Lumbar cortico-spinal tract | 12,900 ± 1,100 | 13,200 ± 1,700 | 11,400 ± 3,000 |
| Nucleus of Onufrowicz | 11,100 ± 900 | 10,900 ± 3,000 | 12,400 ± 1,200 |

Data are presented as mean ± SD (U/g wet weight). No significant differences between controls, SALS and FALS cases were found for any of the CNS areas by analysis of variance (ANOVA).
