# Supplementary material for: Novel Antibodies Reveal Inclusions Containing Non-Native SOD1 in Sporadic ALS Patients
Source: PLoS One. 2010 Jul 14;5(7):e11552. doi: 10.1371/journal.pone.0011552 (PMC2904380; doi:10.1371/journal.pone.0011552)
Supplement: Table S2 — SOD2 and SOD3 activities in different CNS areas from controls, SALS and FALS patients. (0.05 MB DOC) [file pone.0011552.s009.doc]

| **Table S2.** SOD2 and SOD3 activities in different CNS areas from controls, SALS and FALS patients. | | | | | | |
| --- | --- | --- | --- | --- | --- | --- |
|  | Controls (n=5) | | SALS (n=5) | | FALS (n=4) | |
| CNS area | SOD2 | SOD3 | SOD2 | SOD3 | SOD2 | SOD3 |
| Frontal lobe | 1,060 ± 300 | 88 ± 41 | 1,020 ± 130 | 84 ± 21 | 760 ± 270 | 87 ± 12 |
| Temporal lobe | 880 ± 120 | 89 ± 27 | 830 ± 130 | 88 ± 26 | 870 ± 320 | 102 ± 26 |
| Precentral gyrus | 930 ± 380 | 101 ± 33 | 920 ± 120 | 70 ± 14 | 1,070 ± 620 | 107 ± 4 |
| Corona radiata | 290 ± 80 | 47 ± 14 | 380 ± 260 | 42 ± 11 | 330 ± 170 | 47 ± 12 |
| Ventral vermis | 1,090 ± 270 | 78 ± 41 | 1,070 ± 270 | 76 ± 20 | 980 ± 340 | 111 ± 88 |
| Lamina tecti | 1,210 ± 690 | 148 ± 142 | 1,270 ± 40 | 79 ± 26 | 1,030 ± 560 | 106 ± 58 |
| Motor nuclei | 680 ± 240 | 81 ± 38 | 830 ± 260 | 87 ± 46 | 870 ± 210 | 241 ± 277 |
| Cervical ventral horn | 450 ± 50 | 150 ± 47 | 730 ± 460 | 114 ± 53 | 680 ± 120 | 152 ± 84 |
| Cervical dorsal funicle | 240 ± 50 | 129 ± 55 | 290 ± 150 | 138 ± 78 | 560 ± 550 | 192 ± 78 |
| Cervical cortico-spinal tract | 280 ± 100 | 115 ± 133 | 820 ± 210 | 162 ± 100 | 810 ± 780 | 96 ± 38 |
| Lumbar ventral horn | 720 ± 170 | 87 ± 35 | 810 ± 270 | 124 ± 82 | 790 ± 420 | 86 ± 34 |
| Lumbar dorsal horn | 540 ± 60 | 98 ± 58 | 460 ± 100 | 154 ± 116 | 550 ± 140 | 124 ± 76 |
| Lumbar dorsal funicle | 300 ± 110 | 109 ± 54 | 260 ± 100 | 132 ± 65 | 210 ± 60 | 199 ± 162 |
| Lumbar cortico-spinal tract | 480 ± 90 | 153 ± 65 | 570 ± 160 | 158 ± 101 | 730 ± 340 | 295 ± 104 |
| Nucleus of Onufrowicz | 570 ± 200 | 239 ± 180 | 660 ± 230 | 175 ± 93 | 600 ± 340 | 384 ± 49 |

Data are presented as mean ± SD (U/g wet weight). No significant differences between controls, SALS and FALS cases were found for any of the CNS areas, by analysis of variance (ANOVA).
