# Supplementary material for: Novel Antibodies Reveal Inclusions Containing Non-Native SOD1 in Sporadic ALS Patients
Source: PLoS One. 2010 Jul 14;5(7):e11552. doi: 10.1371/journal.pone.0011552 (PMC2904380; doi:10.1371/journal.pone.0011552)
Supplement: Table S3 — Antibodies used in morphological studies. (0.05 MB DOC) [file pone.0011552.s010.doc]

| **Table S3.** Antibodies used in morphological studies. | | | | | |  |
| --- | --- | --- | --- | --- | --- | --- |
| **Description** | **Product identification** | **Antibody concentration** | | | **Manufacturer** | |
| Primary antibodies | | | | | | |
| Rabbit anti-SOD1^1^ | Rabbit-1 | |  | | In house | |
| Chicken anti-SOD1 |  | |  | | In house | |
| Rabbit anti-KLH^1,2^ |  | | |  | In house | |
| Sheep anti-SOD1 | PC077 | | | 24 µg/ml | The Binding Site, Birmingham, UK | |
| Sheep anti-SOD1 | 574597 | | | 450 µg/ml | Calbiochem, La Jolla, CA, USA | |
| Mouse anti-SOD1 | S-2147 | | | 240 µg/ml | Sigma, Schelldorf, Germany | |
| Rabbit anti-GFAP | Z0334 | | | 0.24 µg/ml | Dako, Glostrup, Denmark | |
| Rabbit anti-Ubiquitin | Z5116 | | | 1.9 µg/ml | Dako, Glostrup, Denmark | |
| Rabbit anti-UCHL-1^3^ | Z0458 | | | 2 µg/ml | Dako, Glostrup, Denmark | |
| Mouse anti-TDP-43^4^ | H0002345-M01 | | | 2 µg/ml | Abnova, Taipei, Taiwan | |
| Mouse anti-Cathepsin-D | ab6313 | | | 10.4 µg/ml | Abcam, Cambridge, UK | |
| Mouse anti-KDEL | ab12223 | | | 5 µg/ml | Abcam, Cambridge, UK | |
| Mouse anti-mitochondrial-Hsp70 | ab2799 | | | 2.5 µg/ml | Abcam, Cambridge, UK | |
| Mouse anti-Ubiquitin | ab7254 | | | 10 µg/ml | Abcam, Cambridge, UK | |
| Mouse anti-Mitochondrial marker | NB120-17857 | | |  | Novus Biologicals, Littleton, CO, USA | |
| Rabbit anti-GRP 78 (H-129) | sc-13968 | | | 4 µg/ml | Santa Cruz Biotechnology, Santa Cruz, CA, USA | |
| Rabbit anti-MAP1LC3A | Ab52628 | | | 1:50 | Abcam, Cambridge, UK | |
| Secondary antibodies | | | | | | |
| Goat anti-rabbit IgG^5^ | A-11008 | | |  | Molecular Probes, Eugene, OR, USA | |
| Goat anti-mouse IgG1^6^ | A-21127 | | |  | Molecular Probes, Eugene, OR, USA | |
| Goat anti-mouse IgG2a^7^ | A-21137 | | |  | Molecular Probes, Eugene, OR, USA | |
| Goat anti-mouse IgG3^8^ | A-21157 | | |  | Molecular Probes, Eugene, OR, USA | |
| Goat anti-chicken IgG^9^ | A-11039 | | |  | Molecular Probes, Eugene, OR, USA | |

^1^ The IgG fraction was isolated with Protein A-Sepharose, ^2^ KLH = Keyhole limpet hemocyanin , ^3^ Ubiquitin carboxyl-terminal hydrolase isozyme L-1, ^4^ TAR DNA-binding protein 43, ^5^ Recognizing the rabbit SOD1 peptide antibodies, ^6^ Recognizing the TDP-43 and mouse anti-Ubiquitin antibodies, ^7^ Recognizing the cathepsin D and KDEL antibodies, ^8^ Recognizing the mitochondrial HSP70 antibody, ^9^ Recognizing the chicken SOD1 peptide antibodies.
